# Both BRCA1-wild type and -mutant triple-negative breast cancers show sensitivity to the NAE inhibitor MLN4924 which is enhanced upon MLN4924 and cisplatin combination treatment

**DOI:** 10.18632/oncotarget.27485

**Published:** 2020-02-25

**Authors:** Shrilekha Misra, Xiaoli Zhang, Nissar Ahmad Wani, Steven Sizemore, Alo Ray

**Affiliations:** ^1^Department of Pathology, The Ohio State University, Columbus, OH, USA; ^2^Department of Radiation Oncology, The Ohio State University, Columbus, OH, USA; ^3^Comprehensive Cancer Center, The Ohio State University, Columbus, OH, USA; ^4^Department of Biomedical Informatics, The Ohio State University, Columbus, OH, USA

**Keywords:** triple-negative breast cancer, MLN4924, cisplatin, cell cycle, DNA damage

## Abstract

Triple-negative breast cancer (TNBC) shows limited therapeutic efficacy. PARP inhibitor has been approved to treat advanced BRCA-mutant breast cancer but shows high resistance. Therefore, the development of new therapeutics that sensitize TNBC irrespective of BRCA status is urgently needed. The neddylation pathway plays a critical role in many physiological processes by regulating the degradation of proteins. MLN4924, a selective inhibitor of the key neddylation enzyme NEDD8 Activation Enzyme (NAE1), shows higher sensitivity to both BRCA1-wild type and -mutant TNBCs compared to other breast cancer subtypes. MLN4924 induced re-replication with >4N DNA content leading to robust DNA damage. Accumulation of unrepaired DNA damage resulted in S and G2/M arrest causing apoptosis and senescence, due to the stabilization of the replication initiation protein CDT1 and the accumulation of cell cycle proteins upon MLN4924 treatment. Moreover, adding MLN4924 to the standard TNBC chemotherapeutic agent cisplatin increased the DNA damage level, further enhancing the sensitivity. *In vivo*, MLN4924 reduced tumor growth in a NOD-SCID mouse xenograft model by inducing DNA damage which was further augmented with the MLN4924 and cisplatin cotreatment. NAE1 is overexpressed in TNBC cell lines and in patients compared to other breast cancer subtypes suggesting that NAE1 status is prognostic of MLN4924 treatment response and outcome. Taken together, we demonstrated the mechanism of TNBC sensitization by the MLN4924 and MLN4924/cisplatin treatments irrespective of BRCA1 status, provided a strong justification for using MLN4924 alone or in combination with cisplatin, and identified a genetic background in which this combination will be particularly effective.

## INTRODUCTION

Triple-negative breast cancer (TNBC) comprises 15–20% of breast cancer. This breast cancer subtype does not express estrogen alpha receptors (ER) or progesterone receptors (PR) and lacks overexpression of the HER2 gene [1]. TNBC shows a high rate of early recurrence, poor prognosis, no response to hormonal therapy, only partial response to radio- and chemo-therapy, and lacks targeted therapy options [1], warranting the development of more effective therapeutics. Recently, Poly (adenosine diphosphate (ADP)-ribose) polymerase inhibitor (PARPi) has been approved to treat metastatic BRCA-mutant breast cancer [2, 3]. BRCA1 and BRCA2 are involved in DNA damage repair by homologous recombination (HR), replication fork stabilization, and maintenance of genomic stability [4, 5]. Mutations in these genes predispose patients to hereditary breast cancer [5]. Unfortunately, resistance to PARPi due to the reversion of BRCA mutations and other mechanisms reduces drug efficacy, and only a fraction of TNBC patients harbor BRCA mutations [6], limiting the efficacy of PARPi-based therapies. Thus, the development of advanced therapies that sensitize TNBC regardless of BRCA mutation status is essential.

The NEDD8 conjugation cascade, neddylation, is a new target of interest for cancer therapy [7–9]. The neddylation pathway is mainly responsible for the regulated degradation of proteins [10] but also plays a role in other physiological processes [11, 12]. NEDD8 is activated by the E1 enzyme, NEDD8 activation enzyme (NAE1), comprised of NAE1/APPBP1 and UBA3 heterodimer in an ATP-dependent manner. Activated NEDD8 is then transferred to an ubiquitin-conjugating enzyme UBE2M/UBC12 (E2). E2 collaborates with the E3 ligase, cullin-RING ubiquitin ligases (CRLs), and conjugates NEDD8 to CRLs [13]. Activated CRL-mediated ubiquitination of substrate proteins targets them for degradation [10] or performs other physiological functions [11, 12] (Supplementary Figure 1A). The CRLs control the degradation of many proteins involved in DNA replication, DNA damage response, cell cycle progression, apoptosis, and senescence [14–16]. MLN4924 (MLN), a selective small-molecule NAE1 inhibitor, is being evaluated to treat numerous cancers and is in Phase I clinical trials [17]. MLN4924 binds to the NAE1 active site blocking neddylation and the downstream pathway (Supplementary Figure 1A), causing the accumulation of CRLs and their substrates [16]. MLN4924 sensitivity and mechanism of function are not well characterized across breast cancer subtypes. Recent studies showed that MLN4924 sensitizes the ER^+^ BRCA1-wild type breast cancer cell lines to radiation [18]. However, whether MLN4924 sensitizes breast tumors *in vivo*, is cytotoxic to BRCA1-mutated TNBC, and sensitizes TNBC to standard TNBC chemotherapeutics have not been investigated. Here, we evaluated the efficacy of MLN4924 as a therapeutic agent in BRCA1-wild type and -mutant cells and examined if MLN4924 in combination with cisplatin (Cis), a standard platinum-based TNBC chemotherapeutic, enhances cytotoxicity. Platinum-based regimens showed higher sensitivity in TNBC compared to non-TNBC patients, and recently there has been a renewed interest for platinum therapy in TNBC [19, 20]. Therefore, we examined if combining MLN4924 with cisplatin will increase the therapeutic efficacy of TNBC treatment.

Cisplatin triggers intra- and inter-strand crosslinking of DNA by forming platinum-DNA adducts/crosslinks [20, 21]. The initial incision of the cross-link requires nucleotide excision repair pathway enzymes [22], which process the ICLs into double-strand breaks (DSBs). These DSBs are repaired by the Fanconi anemia (FA) and HR repair pathways [23]. Additionally, upon DNA damage, ATR and ATM kinases activate the cell cycle checkpoint by phosphorylating Chk1 and Chk2 respectively, allowing time to repair the DNA [24]. ATM and ATR also phosphorylate H2AX on S139 at the sites of DNA damage and replication stress which helps recruit other repair and checkpoint proteins [24, 25]. ATR and ATM also phosphorylate BRCA1 which plays a key role in DSB repair by HR, S phase checkpoint, and G2/M phase checkpoint [4, 26].

Our results demonstrated that TNBC cell lines show a higher sensitivity to MLN4924 compared to cell lines representing other breast cancer subtypes due to the overexpression of NAE1 in TNBC compared to non-TNBC subtypes. Importantly, both BRCA1-wild type and -mutant cells show sensitivity to MLN4924. MLN4924 treatment resulted in >4N DNA content due to the re-replication of DNA leading to the accumulation of DNA damage, apoptosis, and senescence. Accordingly, MLN4924 triggered the stabilization of the DNA replication initiation factor CDT1 and displayed an accumulation of cell cycle proteins resulting in cell cycle arrest. Notably, the addition of MLN4924 to cisplatin showed enhanced cytotoxicity by increasing the DNA damage level. *In vivo*, MLN4924 significantly inhibited the growth of a TNBC xenograft model by inducing DNA damage, and MLN4924/cisplatin combination further reduced tumor growth by enhancing DNA damage. Combined, these results demonstrate that, unlike PARPi, MLN4924 induces cytotoxicity in both BRCA1-wild type and -mutant TNBC and promotes the efficacy of the cisplatin treatment in a BRCA1-independent manner.

## RESULTS

### MLN4924 exhibits an enhanced antiproliferative effect on both BRCA1-wild type and -mutant TNBCs and shows higher expression of NAE1 compared to other breast cancer subtypes

**Figure 1:**
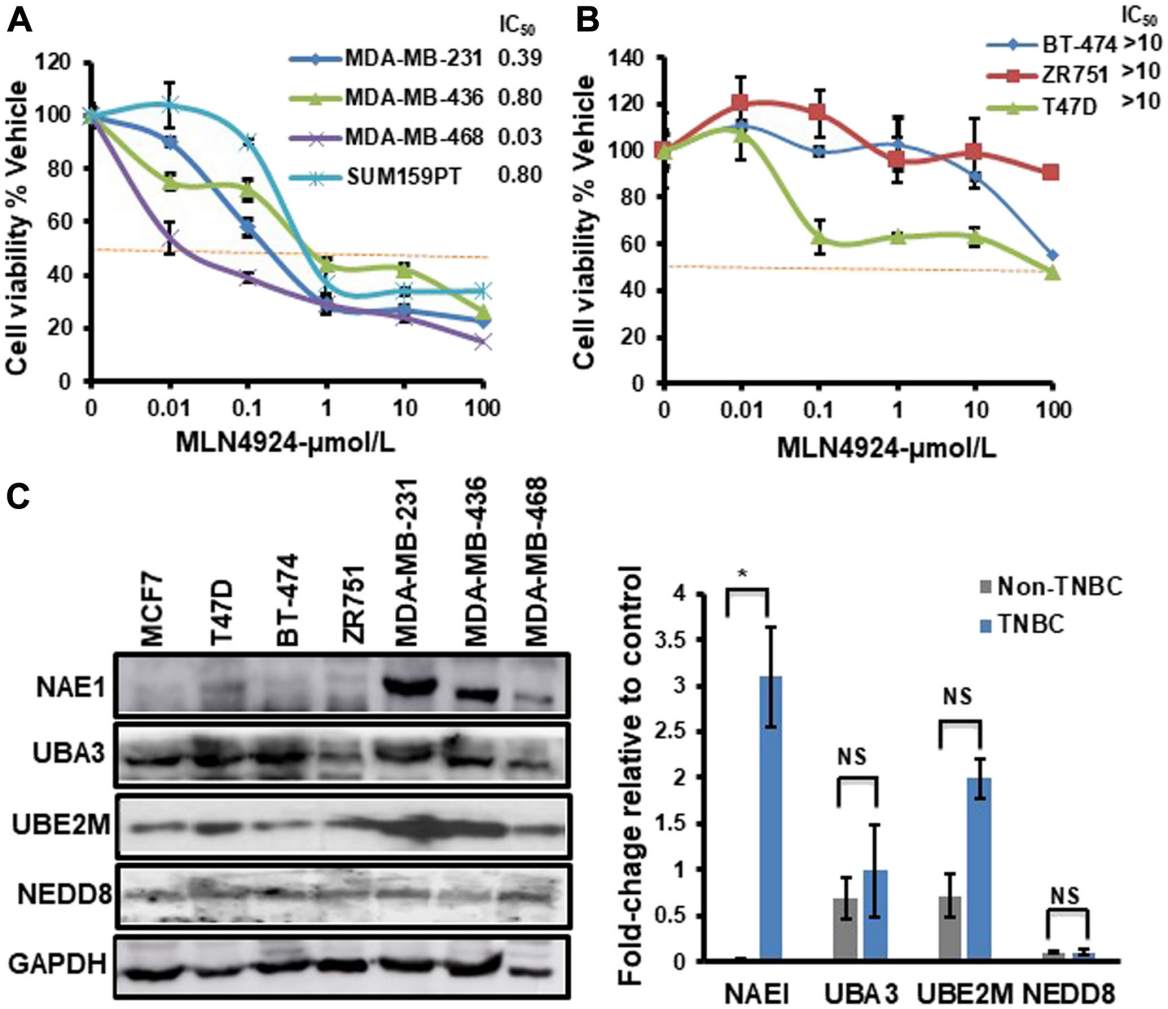
TNBC cells show increased sensitivity to MLN4924 compared to non-TNBC cells and overexpress NAE1. The Cell viability of breast cancer cell lines treated with MLN4924 (µmol/L) as indicated for 86 h was determined by the CellTitre-Glow Luminescent Cell Viability Assay. The cell viability was calculated relative to the DMSO control. Data are expressed as means ± SD of at least three independent experiments. (**A**) TNBC cells. (**B**) Non-TNBC cells. (**C**) WB showing the expression of neddylation pathway proteins in TNBC and non-TNBC cells. WB was quantitated for NAE1, UBA3, UBE2M, and NEDD8 relative to GAPDH control using the Image J software for each cell line. The right panel shows the difference in the expression level of NAE1, UBA3, UBE2M, and NEDD8 in the TNBC group (MDA-MB-231, MDA-MB-436, and MDA-MB-468) compared to the non-TNBC group (MCF7, T47D, BT-474, and ZR-751). Data are expressed as means ± SD between all TNBC (3) and non-TNBC (4) cell lines. ^*^ *p* < 0.05 indicates a significant difference, NS indicates non-significant.

**Figure 2:**
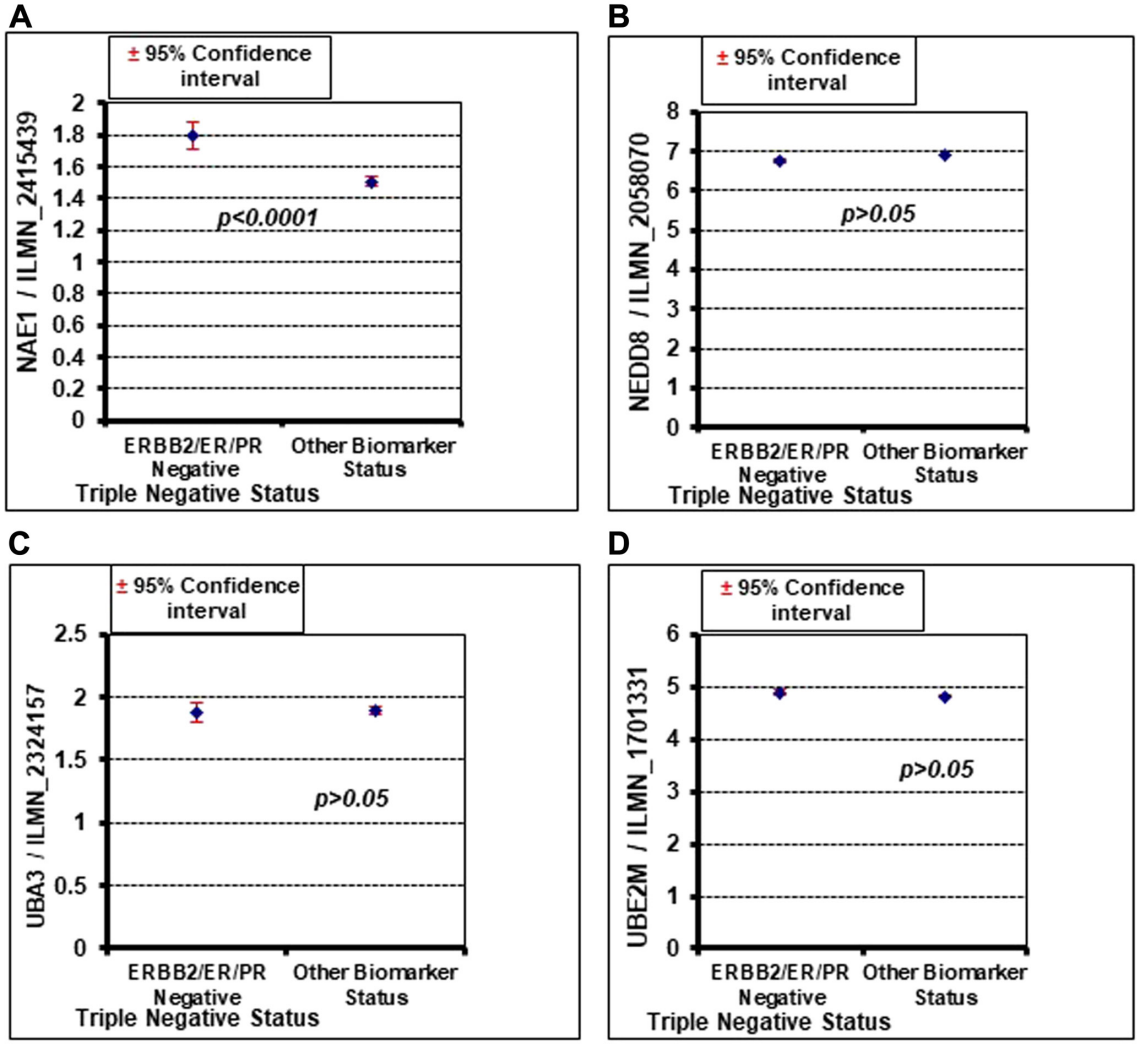
Oncomine database analysis shows that NAE1 is significantly overexpressed in TNBC (ERBB2/ER/PR negative) compared to other breast cancer subtypes (other biomarker status), but NEDD8, UBA3, and UBE2M do not show a significant difference. (**A**) NAE1. (**B**) NEDD8. (**C**) UBA3. (**D**) UBE2M.

The ability of MLN4924 to reduce cell viability was analyzed in a panel of breast cancer cell lines representing different breast cancer molecular subtypes. While MLN4924 reduced the viability of all cell lines tested in a dose-dependent manner, the TNBC cell lines (MDA-MB-231, MDA-MB-436, MDA-MB-468, and SUM159PT) were significantly more sensitive to MLN4924 (average IC_50_ <0.50 µmol/L) than the non-TNBC cell lines (BT-474, ZR751, and T47D; average IC_50_>10 µmol/L) (Figure 1A, 1B). At 1 µM MLN4924, almost all TNBC cell lines displayed >50% cell death (Figure 1A). Thus, MLN4924 as a single agent is a potent inhibitor of TNBC cell viability. We used both BRCA1 wild type (MDA-MB-231, MDA-MB-468, and SUM159PT) and BRCA1-mutated (MDA-MB-436) TNBC cell lines and observed that, as opposed to PARP inhibitor, both BRCA1-wild type and mutant cell lines show equal sensitivity to MLN4924 (All IC_50_ below 1 µM, Figure 1A). Given that MLN4924 is a specific inhibitor of NAE1, we evaluated the expression of the neddylation enzymes by Western blot (WB) assay in TNBC and non-TNBC cell lines and found that NAE1 is overexpressed in the TNBC group compared to the non-TNBC group (*p* < 0.01) while UBA3, UBE2M, and NEDD8 did not show significant differences between these two groups (*p* > 0.05) (Figure 1C). Additionally, analyses of a publicly available breast cancer patient gene expression database confirmed that NAE1 is overexpressed in TNBC patient samples compared to non-TNBC samples (*p* < 0.0001) while UBA3, UBE2M, and NEDD8 expression did not show a significant difference (*p* > 0.05) (Figure 2A–2D). These results indicate that overexpression of NAE1 might be an important determinant of increased MLN4924 cytotoxicity in TNBC, and MLN4924 could serve as an attractive anticancer drug for TNBC irrespective of BRCA1 status.

### MLN4924 enhances the cytotoxicity of both BRCA1-wild type and -mutant TNBC cells to cisplatin

**Figure 3:**
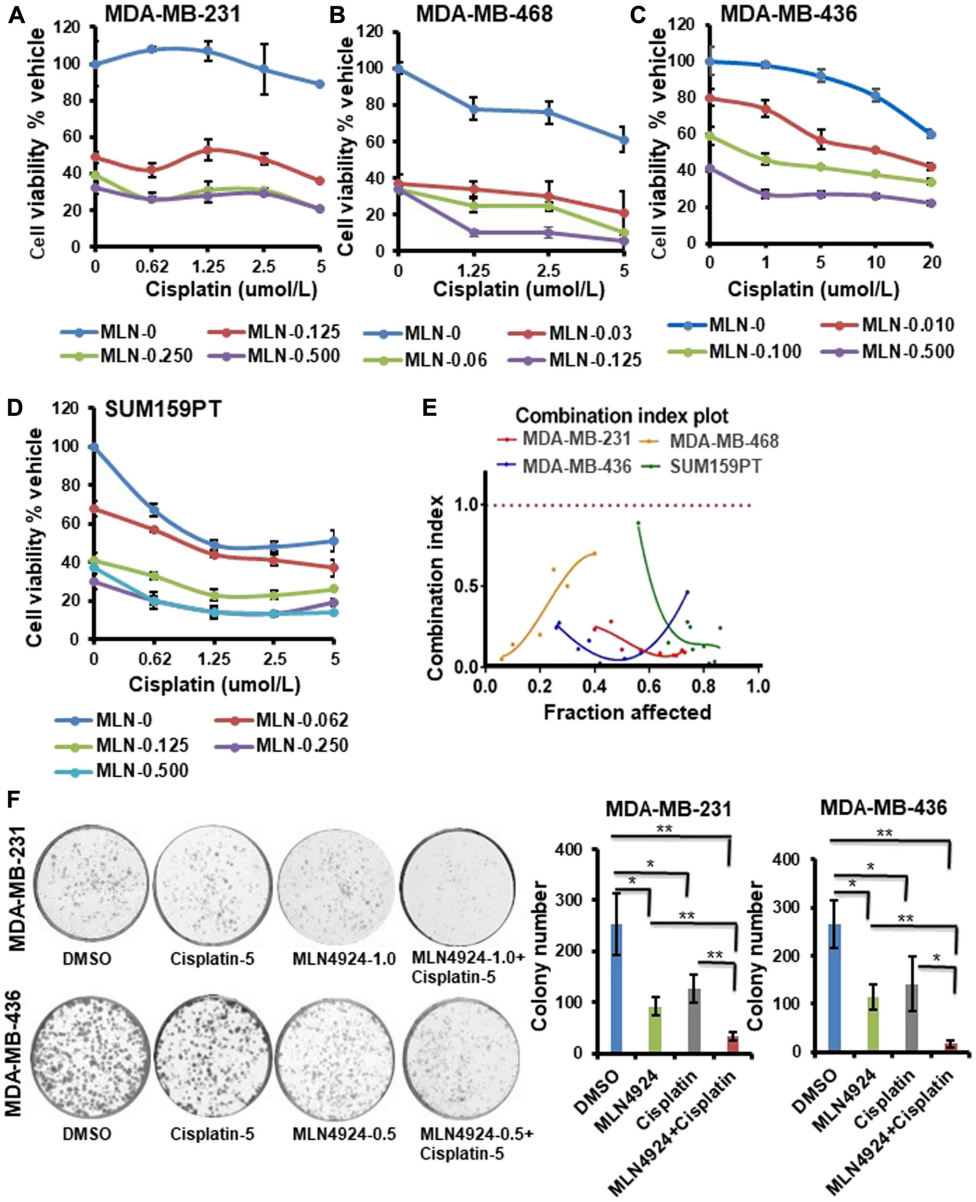
MLN4924 shows enhanced sensitization of both BRCA1- wild type and -mutant TNBC cells when combined with cisplatin. (**A–D**) Clonogenic cell survival of TNBC cell lines treated with MLN4924, cisplatin, or MLN4924/cisplatin at the indicated doses. The x-axis represents the cisplatin doses, and the y-axis represents the cell viability % vehicle of MLN4924 and MLN4924/cisplatin combination. Data are expressed as means ± SD of at least three independent experiments. (**E**) CI of MLN4924 and cisplatin in TNBC cells by the Chou-Talalay method. (**F**) Colony formation assay to show cell death upon treating the cells with MLN4924 (0.5 or 1 µM), cisplatin (5 µM), and MLN4924/cisplatin as indicated. Representative images of three independent experiments are shown. Data are expressed as means ± SD of three independent experiments. ^*^ *p* < *0.01* and ^**^ *p* < *0.001* indicate a significant difference.

Neddylation plays a key role in the modification and degradation of many proteins in DNA damage repair and replication [7, 15, 27]; therefore, we hypothesized that MLN4924 would sensitize cancer cells to DNA damaging chemotherapeutics by inhibiting DNA repair. To investigate, we combined MLN4924 with cisplatin, a platinum-based chemotherapeutic drug for TNBC [19, 20]. First, we determined the IC_50_ of cisplatin (Supplementary Figure 1B) and used cisplatin below the IC_50_ for the combination experiments. The combination treatment augmented the sensitivity in all 4 TNBC cell lines tested (Figure 3A–3D). The synergy between the two drugs was quantified by the combination index (CI) using the Chou Talalay method and the Compusyn software as described in Materials and Methods [28]. The CI for the MLN4924/cisplatin combination is less than 1 in all TNBC cell lines tested (Figure 3E), indicating a synergistic effect. Consistent with this, TNBC cells displayed reduced colony formation efficiency upon MLN4924 treatment in a dose-dependent manner, which was further reduced by combination treatment with cisplatin (*p* < 0.01–0.001 for Figure 3F, Supplementary Figure 1C).

**Figure 4:**
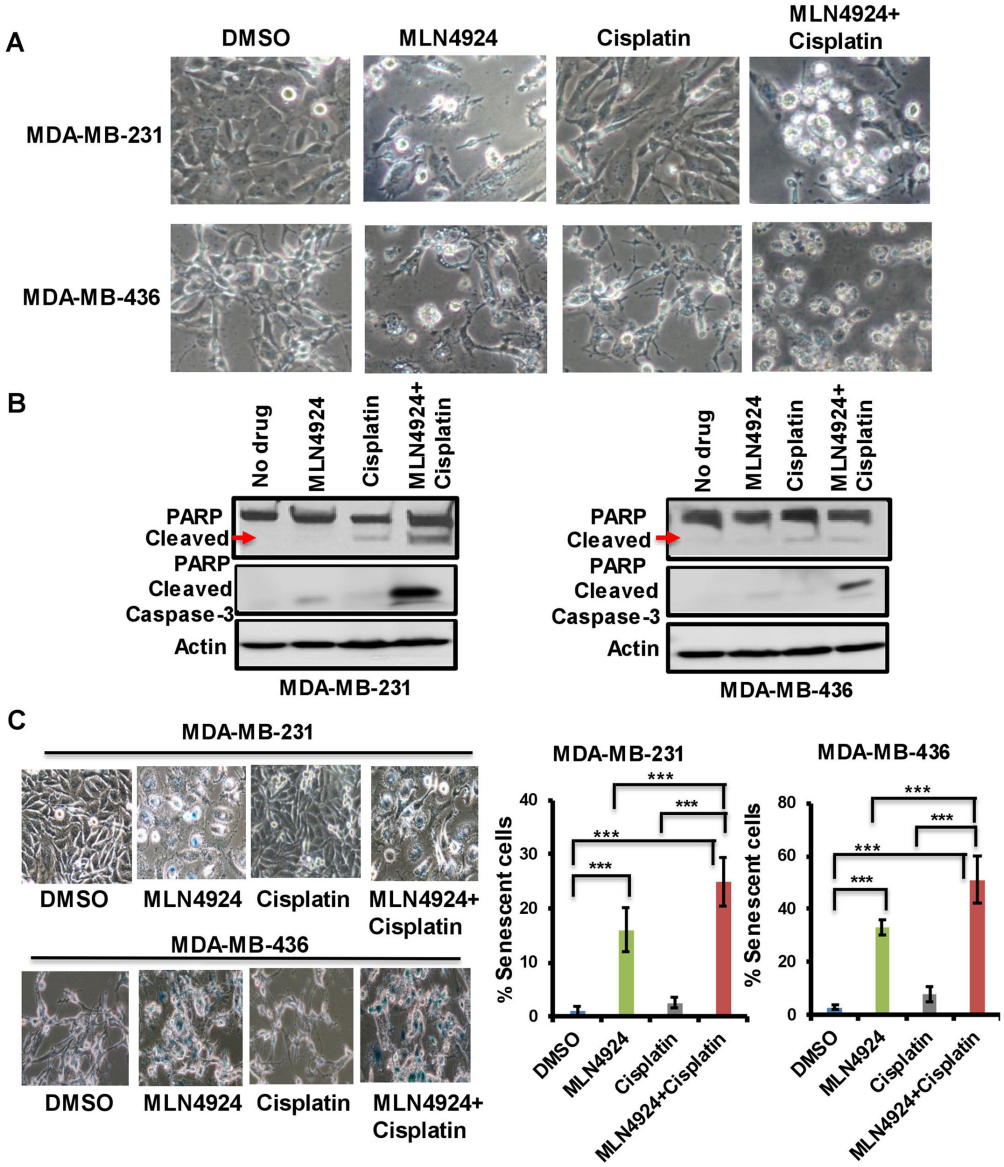
MLN4924 induces apoptosis and senescence in TNBC cells which are enhanced by MLN4924/cisplatin combination treatment. (**A**) Phenotypic changes of MDA-MB-231 and MDA-MB-436 cells upon MLN4924 (2 µM), cisplatin (5 µM), and MLN4924/cisplatin treatments for 48 h as described in Materials and Methods. Pictures were taken at 40× magnification. (**B**) MLN4924 and MLN4924/cisplatin treatments induce apoptosis. Cells were treated with MLN4924 (2 µM), cisplatin (10 µM), and MLN4924/cisplatin for 48 h and WB was performed using PARP and cleaved caspase 3 antibodies. (**C**) MLN4924 induces senescence in TNBC cells. Cells were treated with MLN4924 (2 µM), cisplatin (5 µM), or MLN4924/cisplatin for 48 h. Senescence β-gal assay was performed as described in Materials and Methods. Pictures were taken at 40× magnification. Graphs represent the number of blue colored cells. Several fields of cells (~50–200 cells/field) were counted to obtain the % of blue cells for each experiment. The results are from three independent experiments expressed as mean ± SD. ^***^ *p* < *0.0001* indicates a significant difference.

When cells were treated with MLN4924 (1.0 µM, 48 h), they were flattened and enlarged with membrane blebbing implying growth arrest and apoptosis (Figure 4A). Furthermore, MLN4924-treated cells showed a distinct difference in cell morphology from the cisplatin-treated cells (Figure 4A). Consequently, both cells showed the apoptotic marker, cleaved caspase 3, upon MLN4924 treatment which was enhanced upon MLN4924/cisplatin cotreatment (Figure 4B). Furthermore, even though the cleaved PARP level was low upon MLN4924 treatment, it was enhanced upon MLN4924/cisplatin cotreatment (Figure 4B). To examine if senescence also contributes to growth arrest/cell death upon MLN4924 treatment, we performed SA β-gal staining upon drug treatments. A significant fraction of cells underwent senescence as evident by the SA β-gal staining of the cells (*p* < 0.0001; Figure 4C). Additionally, both cells showed increased senescence upon MLN4924/cisplatin combination treatment compared to MLN4924 and cisplatin alone. Interestingly, MDA-MB-436 showed more senescence (33% MLN4924 and 51% MLN4924/cisplatin) compared to MDA-MB-231 (16% MLN4924 and 25% MLN4924/cisplatin) (Figure 4C) indicating that BRCA1 in MDA-MB-231 might reduce senescence or that this phenotype may be due to a physiological difference between the two cell lines. Nonetheless, these results corroborated that both BRCA1-wild type and -mutant cells undergo apoptosis and senescence at different levels, which were augmented by combination treatment.

### MLN4924 and MLN4924/cisplatin treatments result in extensive re-replication and S phase arrest which are partially dependent on BRCA1

**Figure 5:**
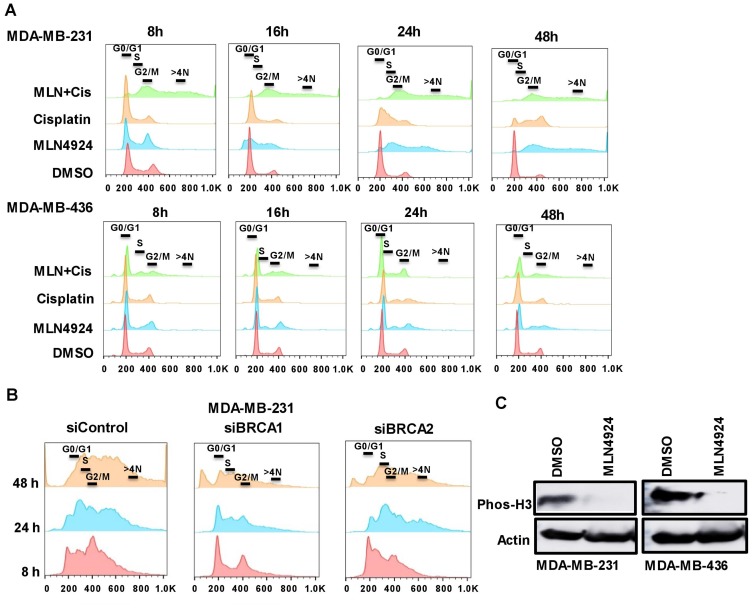
The inhibition of the neddylation pathway results in re-replication and S/G2 arrest which are partially dependent on BRCA1. (**A**) MDA-MB-231 and MDA-MB-436 cells were treated with DMSO, MLN4924 (2 µM), cisplatin (10 µM), and MLN4924/cisplatin for indicated times. DNA profiles were analyzed by flow cytometry. The x-axis represents the DNA area, and the y-axis represents the cell count. The peaks under 600 and 800 represent >4N DNA content. (**B**) BRCA1 plays a role in re-replication and S arrest whereas BRCA2 does not. MDA-MB-231 cells were transfected with BRCA1 and BRCA2 siRNAs for 48 h and left untreated or treated with MLN4924 (2 µM) for the indicated times. DNA profiles were analyzed by flow cytometry. (**C**) The phos-H3 level is low upon MLN4924 treatment indicating that cells do not progress to mitosis. Cells were treated with DMSO and MLN4924 (2 uM) for 48 h and WB was performed.

As MLN4924 induces a cell cycle defect [7], we postulated that the flattened and enlarged cells upon MLN4924 treatment may have been caused by a cell cycle progression defect. Therefore, we assessed the cell cycle profiles by DNA content using flow cytometry. Upon MLN4924 treatment, MDA-MB-231 cells began to accumulate in S with DNA re-replication resulting in >4N DNA content (polyploidy), and consequently, a lower number of cells proceeded to G1. Furthermore, the MLN4924/cisplatin combination significantly increased the polyploidy compared to MLN4924 alone (Figure 5A, Supplementary Figure 2A, 2B). Initially, most of the cisplatin-treated cells were in G1, but they slowly progressed to S. The cell cycle analysis of MDA-MB-468 also showed similar results (Supplementary Figure 3A, 3B). Interestingly, even though MDA-MB-436 (BRCA1-mutant) cells displayed re-replication (>4N DNA), it was significantly lower than that of the MDA-MB-231 (BRCA1-wild type) cells, and a substantial population of cells progressed to G1 (Figure 5A, Supplementary Figure 2A, 2B). By 24 h, only 20% of MDA-MB-436 cells showed >4N DNA content compared to 45–50% MDA-MB-231 cells. Accordingly, ~55% of MDA-MB-231 and ~30% of MDA-MB-436 cells showed BrdU incorporation compared to 14% and 10% of control cells, further supporting that cells accumulated in S phase and continued replication (*p* < 0.0001, Supplementary Figure 4A). We hypothesized that MDA-MB-436 cells would show lower re-replication due to mutated BRCA1 since BRCA1 plays a role in re-replication, S arrest, and G2/M arrest [29]. To investigate this, we knocked down BRCA1 in MDA-MB-231 cells (Supplementary Figure 4B) and assessed the cell cycle progression and polyploidy after drug treatments. Upon 24 h MLN4924 treatment, 20% of BRCA1-depleted MDA-MB-231 cells displayed polyploidy compared to 32% of control cells (Figure 5B, Supplementary Figure 4C, 4D). At 48 h post-treatment, 27% of BRCA1-depleted cells showed polyploidy compared to 43% of control cells (Figure 5B, Supplementary Figure 4C, 4D). Accordingly, 21% BRCA1-depleted MDA-MB-231 cells progressed to G1 whereas 9% control cells progressed to G1 after 48 h of MLN4924 treatment. Thus, the reduction in re-replication and S phase cells in BRCA1-mutant cells is at least partially due to the loss of BRCA1 function. As BRCA2 functions with BRCA1 in the HR pathway [30], we examined if BRCA2 depletion shows the same defect. Upon 48 h of MLN4924 treatment, 48% of BRCA2-depleted cells showed polyploidy similar to 44% control cells, and 8% of BRCA2-depleted cells were in G1 similar to 8% control cells (Figure 5B, Supplementary Figure 4C, 4D), showing that BRCA2 knockdown did not rescue the polyploidy. Consistent with the cell cycle progression defects, the mitotic marker phos-H3 was reduced by both WB and immunofluorescence (IF) assays (Figure 5C, Supplementary Figure 5A). Upon 48 h of MLN4924 treatment, 1% of MDA-MB-231 and 4% of MDA-MB-436 cells showed phos-H3 foci compared to ~12–19% control cells further validating that cells could not proceed to mitosis. Collectively, both BRCA1-wild type and -mutant cells exhibited re-replication and accumulation of cells in S phase following MLN4924 and MLN4924/cisplatin treatments, but this effect is more pronounced in BRCA1-wild type cells.

### MLN4924 and MLN4924/cisplatin treatments induce accumulation of proteins in DNA replication and cell cycle pathways

**Figure 6:**
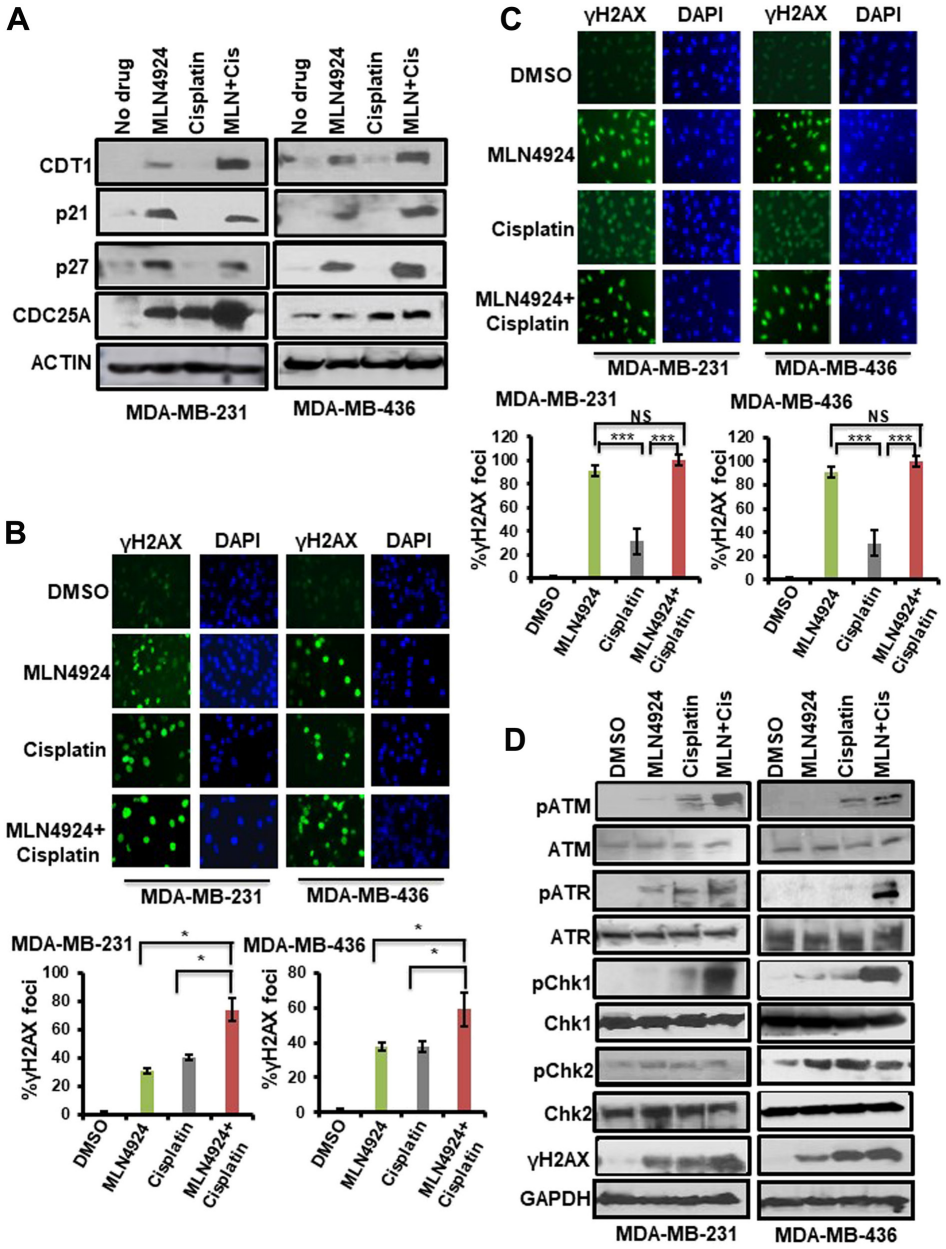
Inhibition of neddylation pathway induces accumulation of neddylation substrates, DNA damage, and the activation of the cell cycle checkpoint which are enhanced upon MLN4924/cisplatin cotreatment. (**A**) WB showing the accumulation of CRL-substrates upon MLN4924, cisplatin, and MLN4924/cisplatin treatments. Cells were treated with MLN4924 (2 µM), cisplatin (10 µM), and MLN4924/cisplatin for 6 h. Cell lysates were immunoblotted with CRL substrates. (**B**) Induction of DNA damage was monitored by γH2AX foci formation by IF after treating the cells with MLN4924 (2 µM), cisplatin (10 µM), and MLN4924/cisplatin for 6 h. The top panel shows representative fields from one experiment. IF pictures were taken at 40× magnification; the results were quantitated from 3 independent experiments, and for each experiment, at least 3–5 independent fields were counted. Data was presented as mean ± SD. ^*^ *p* < *0.01* indicates a significant difference. (**C**) Cells were treated with MLN4924 (2 µM), cisplatin (10 µM), and MLN4924/cisplatin for 12 h. The cells were washed and allowed to post-repair for 12 h, and DNA damage was monitored by γH2AX foci formation. The top panel shows the representative fields from one experiment. The experiment was done as described in (B). Data was presented as mean ± SD. ^***^ *p* < *0.0001* indicates a significant difference. *NS* indicates non-significant. (**D**) WB showing the DNA damage by γH2AX level and cell cycle checkpoint activation by phosphorylation of ATR, ATM, Chk1, and Chk2. Cells were treated with MLN4924 (2 µM), cisplatin (10 µM), and MLN4924/cisplatin for 24 h. Cell lysates were immunoblotted with specific antibodies.

Since MLN4924 and MLN4924/cisplatin treatments showed re-replication and cell cycle defects [7], we analyzed the levels of CRL substrates regulating these pathways. MLN4924 inhibits Cullin 1 and 4 neddylation and subsequently accumulates replication initiation protein CDT1, which induces DNA re-replication and collision of replication forks leading to DNA damage [31]. Consequently, CDT1 inhibits cell proliferation and displays an important effect on MLN4924 anti-cancer activity [7]. We also analyzed the accumulation of CDC25A, p21, and p27 CRL substrates inducing cell cycle arrest [7, 15]. As expected, MLN4924 and MLN4924/cisplatin treatments led to a robust accumulation of these proteins in both BRCA1-wild type and -mutant TNBC cells (Figure 6A). These data demonstrated that CDT1 stabilization leads to DNA re-replication resulting in robust >4N DNA content, replication stress, and DNA damage which are predominant phenotypes upon MLN4924 treatment (Figure 5A, Supplementary Figures 2, 3, 4A). The robust DNA damage results in S and G2/M arrest which were also influenced by the accumulation of p21, p27, and Cdc25A. Thus, these pathways largely contributed to cell death. As p21 plays a role in senescence [32], we predict that p21 accumulation upon MLN4924 treatment (Figure 6A) may contribute to senescence.

### MLN4924 promotes DNA damage and activates the cell cycle checkpoint which are enhanced upon MLN4924/cisplatin cotreatment

Replication stress and DNA damage induce H2AX phosphorylation at S139, which promotes accumulation of DNA damage repair and cell cycle checkpoint proteins at the replication stress and DNA damage sites [24, 25]. Therefore, we evaluated H2AX phosphorylation (S139) using WB and IF assays. Upon 6 h drug treatment, ~31–38% of MLN4924-treated and ~38–40% of cisplatin-treated cells displayed γH2AX foci (Figure 6B, Supplementary Figure 5B). Notably, MLN4924/cisplatin cotreatment markedly increased the level and numbers of γH2AX foci (59–74%) compared to MLN4924 and cisplatin alone (*p* < 0.01; Figure 6B). To examine the kinetics of DSB repair, we treated the cells with MLN4924 for 12 h and then allowed them to repair damage for an additional 12 h after the treatment period. Almost all cells treated with MLN4924 or MLN4924/cisplatin showed bright γH2AX foci while cells treated with only cisplatin showed 31% γH2AX foci (*p* < 0.0001; Figure 6C). These results demonstrate that cisplatin-induced damage is partly repaired with time whereas MLN4924-induced damage is not, which was also evident by the continued presence of >4N DNA content at 48 h (Figure 5A). In WB assay, the γH2AX level was low at 6 h which increased upon 24 h MLN4924 treatment showing a slow accumulation of DSBs (Supplementary Figure 5C, Figure 6D). As expected, MLN4924/cisplatin treatment significantly enhanced the γH2AX level (Figure 6D). Similarly, MDA-MB-468 cells also showed an increased γH2AX level at 24 h which was augmented by the MLN4924/cisplatin cotreatment (Supplementary Figure 5D). We further confirmed DNA damage by Rad51 foci formation, a marker for HR, upon MLN4924 treatment in BRCA1-and HR-proficient MDA-MB-231 cells (Supplementary Figure 5E). These findings indicate that MLN4924 resulted in a slow accumulation of unrepaired DNA damage, and MLN4924/cisplatin intensified the DNA damage level.

To evaluate the cell cycle checkpoint, we measured the phosphorylation of checkpoint proteins ATR, ATM, Chk1, and Chk2 upon MLN4924 and MLN4924/cisplatin treatments. Both the ATR-Chk1 and ATM-Chk2 pathways showed low activation by phosphorylation upon MLN4924 treatment, but the activation was enhanced by the MLN4924/cisplatin cotreatment (Figure 6D). Interestingly, Chk1 showed significantly enhanced phosphorylation upon MLN4924/cisplation cotreatment whereas Chk2 did not show this enhancement, suggesting that the ATR-Chk1 pathway plays a predominant role in checkpoint activation upon MLN4924/cisplatin cotreatment.

### MLN4924 enhances the anti-tumor effect of cisplatin in xenograft mice model

**Figure 7:**
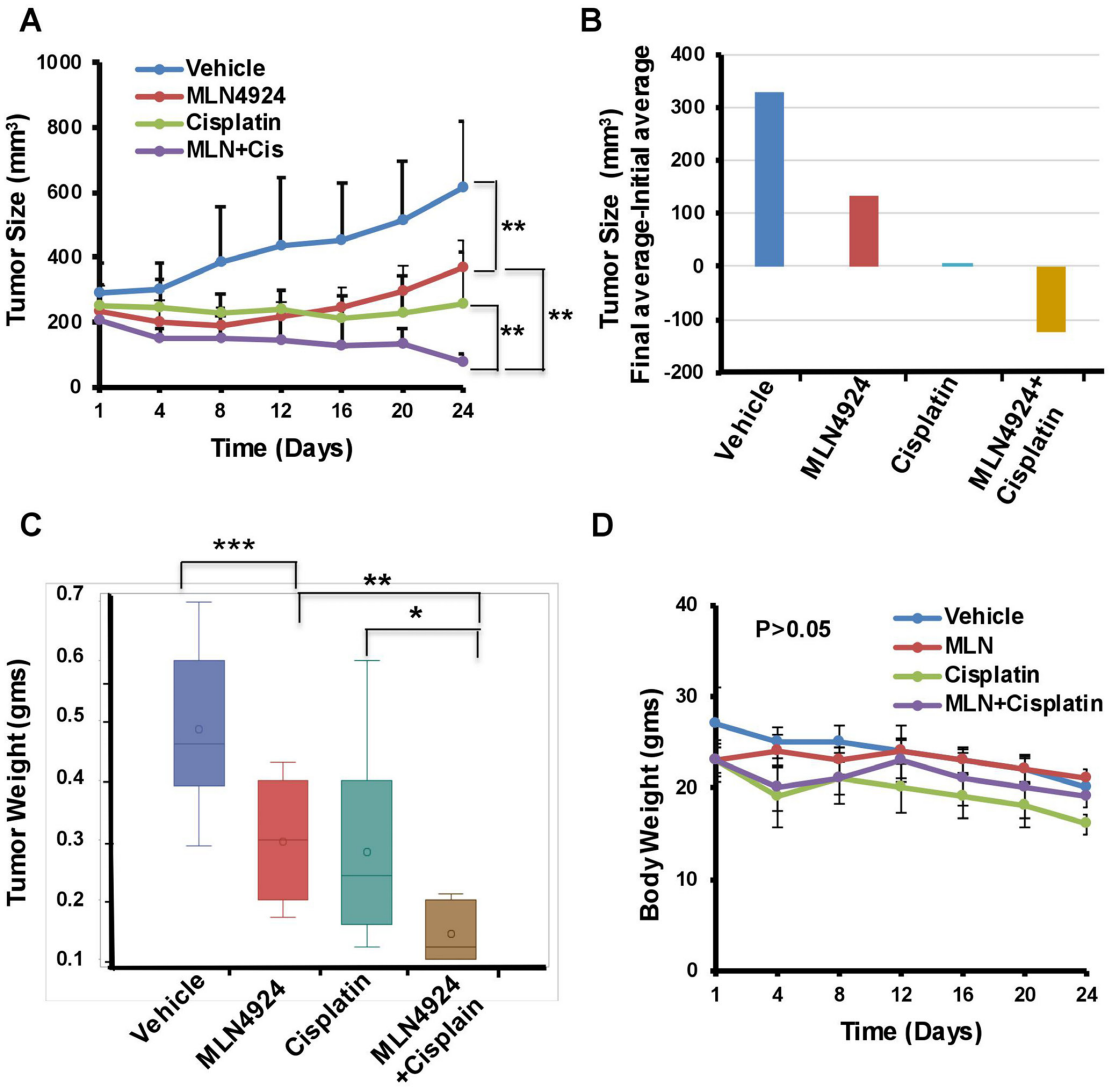
MLN4924 and cisplatin inhibit growth and MLN4924/cisplatin reduces the size of TNBC xenograft tumors. Mice with established subcutaneous tumors were divided into four groups and treated with vehicle, MLN4924, cisplatin, and in combination as described in Materials and Methods (*n* = 11 mice/group). (**A**) Tumor size/volume. (**B**) The difference between the average of initial tumor size and final tumor size. (**C**) Tumor weight. (**D**) The body weight of mice. Two-way Anova was done to determine the *p* values. ^*^ *p* < 0.01, ^**^ *p* < 0.001, and ^***^ *p* < 0.0001 indicate a significant difference. *NS* indicates non-significant.

**Figure 8:**
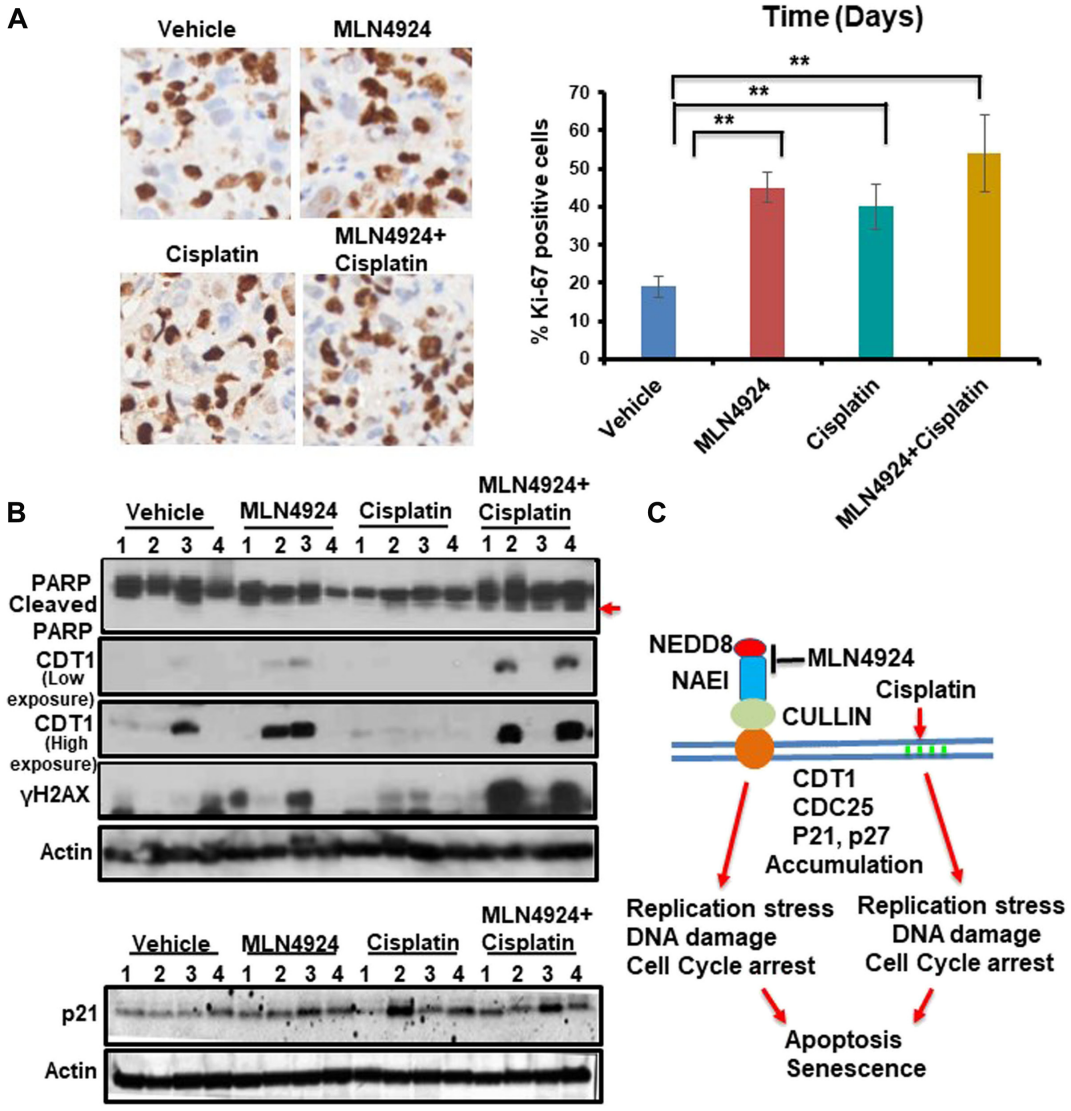
MLN4924 treatment induces DNA damage by stabilizing CDT1 and accumulates the cells in S phase which are enhanced by MLN4924/cisplatin co-treatment. (**A**) Representative images of Ki-67 staining (40× magnification) and quantitation of Ki-67 IHC. The results were from 4 tumors and data were presented as mean ± SD. ^**^ *p* < *0.001* indicates a significant difference. (**B**) WB showing the levels of cleaved PARP, CDT1, γH2AX and p21 in vehicle, MLN4924, Cisplatin, and MLN4924/Cisplatin treated tumors. (**C**) A model showing the pathway leading to apoptosis and senescence upon NAE1 inhibition by MLN4924 and synthetic lethality upon MLN4924/cisplatin cotreatment.

The *in vivo* anti-tumor effect of MLN4924 and cisplatin alone and in combination was evaluated in a NOD-SCID xenograft mouse model using BRCA1-wild type MDA-MB-231 cells. We used BRCA1-wild type cells since BRCA1-mutated cells are generally more sensitive to DNA damaging drugs including cisplatin compared to BRCA1-wild type cells [33, 34]. Therefore, if MLN4924 shows sensitivity to BRCA1-wild type tumors, it will also be effective for BRCA1-mutated tumors. Cells were injected subcutaneously into the flanks of NSG mice. When the tumor size was ~150 mm^3^, mice were divided into four treatment groups: vehicle, MLN4924 (30 mg/kg subcutaneously) [35], cisplatin (2 mg/kg for 3 days, followed by 1.5 mg/kg for 2 days, and 1.0 mg/kg for 2 days (intraperitoneally) [36], and MLN4924/cisplatin combination. MLN4924 was injected 6 h before the cisplatin injection. Tumor size was measured every fourth day and the tumor volume was calculated as described in Materials and Methods (Figure 7A, 7B). After 3 weeks, tumors were harvested, photographed, weighed, and stained with Hematoxylin and Eosin (H&E) to examine the morphology (Figure 7C, Supplementary Figure 6A). MLN4924 or cisplatin alone significantly slowed tumor growth relative to the control (*p* < 0.01–0.0001; Figure 7A–7C). Importantly, coadministration of MLN4924 and cisplatin further reduced tumor volume relative to either treatment alone and resulted in shrinkage of tumors (Figure 7A–7C, Supplementary Figure 6B). None of these treatments resulted in a significant reduction in mouse body weight (*p* > 0.05), suggesting that they were all equally tolerable (Figure 7D). We observed a significantly higher fraction of Ki67, an S phase marker, staining in MLN4924 (45%)- and MLN4924/cisplatin (55%)-treated cells compared to vehicle (20%)-treated cells, supporting our hypothesis that accumulation of S phase cells would result in senescence and apoptosis (Figure 8A). In agreement, MLN4924 induced γH2AX and cleaved PARP in ~50% tumors and even more in tumors treated with both drugs (Figure 8B). Consistent with *in vitro* results, ~50% of tumors from mice treated with MLN4924 or MLN4924/cisplatin demonstrated robust CDT1 levels. Interestingly, we did not observe a substantial enhancement in p21 stabilization *in vivo* with any treatment paradigm (Figure 8B) which suggests that p21 stabilization might be an early event after drug treatment and not visible in tumors after longer time.

## DISCUSSION

The present study demonstrated that MLN4924 would be a potentially successful future therapy for TNBC irrespective of their BRCA1 status. MLN4924 increased the sensitization of both BRCA1-wild type and -mutant TNBCs to cisplatin as opposed to PARPi which showed efficacy for only BRCA1-mutant TNBC. Therefore, combining MLN4924 with cisplatin will have greater therapeutic efficacy for a wider group of TNBC patients. Intriguingly, we found that NAE1 is overexpressed (*p* < 0.0001) in TNBC compared to the non-TNBC group (Figure 1C, Figure 2A) providing a rationale for the increased sensitivity of TNBC to MLN4924 compared to the non-TNBC group. Thus, overexpression of NAE1 in TNBC compared to non-TNBC patients might lead to a novel biomarker identification for TNBC MLN4924 treatment and patient outcome.

Our results demonstrated that both *in vitro* and *in vivo*, MLN4924 induces stabilization of replication initiation factor CDT1 (Figures 6A, 8B) resulting in DNA re-replication with >4N DNA content (Figure 5A) which has been observed in a few other cancer models [7, 31]. Strikingly, we revealed that even though re-replication- and DNA damage-induced cell death occur in both BRCA1-wild type and -mutant cells, it is more dramatic in BRCA1-wild type cells (Figure 5A). We postulate that as BRCA1 is an important player in DNA re-replication and activation of S and G2/M checkpoint [29], these processes are affected in BRCA1-mutant cells. In accordance, a higher fraction of BRCA1-wild type cells showed irreversible S arrest and apoptosis than BRCA1-mutant cells whereas a higher fraction of BRCA1-mutant cells progressed to G1 undergoing senescence (Figures 3F, 5A, 5B, Supplementary Figure 2A, 2B). As shown by others [4, 26], our results supported a role of BRCA1 in re-replication and S/G2 arrest since BRCA2 did not show this effect (Figure 5B). Re-replication leads to DNA damage, and therefore, MLN4924 induced γH2AX foci which were significantly augmented upon MLN4924/cisplatin cotreatment due to additional DNA damage induced by cisplatin (Figure 6B, 6C, 6D). Moreover, the presence of γH2AX upon 12 h post-repair (Figure 6C) and the continuous presence of >4N DNA content (Figure 5A, 5B, Supplementary Figure 2A, 2B) suggest that the extensive DNA damage is not repaired and functions as the driving force for cell death. Even though we observed p21 accumulation in cell lines upon MLN4924 treatment, we did not notice p21-mediated G1 arrest and p21 accumulation in tumors. However, as p21 induces senescence [32], p21 might influence senescence specifically in BRCA1-mutant cells with a higher percentage of G1 cells (Supplementary Figure 2B). Considered together, we envisage that CDT1 accumulation and re-replication plays a predominant role to induce replication stress, DNA damage, and irreversible S arrest upon MLN4924 treatment. Consequently, we observed a delayed cell cycle checkpoint activation probably due to the slow accumulation of replication stress and DNA damage upon MLN4924 treatment, but the cells were unable to repair the extensive DNA damage as evidenced by the presence of unrepaired damage after 12 h and 24 h post-repair (Figure 6C, 6D). Additionally, our results clearly demonstrated that the addition of cisplatin will induce more replication stress and DNA damage, making the cells hyper-reliant on DNA repair and cell cycle checkpoint pathways. Since the ATR-Chk1 pathway is primarily activated in re-replication [29, 37], we found significantly higher ATR and Chk1 activation upon MLN4924 and MLN4924/cisplatin treatments (Figure 6D). Based on these results, we proposed a model showing that the inhibition of neddylation pathway results in accumulation of CDT1 and cell cycle proteins leading to replication stress, DNA damage, and cell cycle arrest causing cell death, which is significantly enhanced upon adding more DNA damage by cisplatin (Figure 8C).

PARPi promotes sensitivity by inducing more DNA damage in BRCA-mutant carriers due to the defective HR but is not very effective for BRCA-wild type breast cancer [38, 39]. Moreover, several studies demonstrated PARPi resistance reducing the efficacy of treatment [6]. Therefore, MLN4924 promoting cytotoxicity for both BRCA1-wild type and -mutant TNBC has a greater potential for a wider group of TNBC patients. Furthermore, since MLN4924 de-regulates S phase replication, it is expected to be more effective for TNBC with an aggressive and faster-growing phenotype compared to non-TNBC patients. Nonetheless, these results suggest that the increased cytotoxicity by MLN4924/cisplatin combination will provide greater cell death with a lower amount of platinum agents. Remarkably, the overexpression of NAE1 in TNBC suggests that NAE1 may function as a biomarker for MLN4924 treatment response of TNBC. Additionally, the presence of p53 mutation in TNBC cells may promote re-replication upon MLN4924 treatment since activation of p53 by ATM/ATR/Chk2 regulates the re-replication through the induction of p21 [40] supporting that p53 status is prognostic of outcome and MLN4924 treatment response of TNBC. Collectively, our results established the molecular mechanism by which MLN4924 induces TNBC cell death and enhances cisplatin sensitivity, provided the rationale of combining MLN4924 with cisplatin in both BRCA1-wild type and mutant TNBCs, and identified a cancer genetic background where this combination will be more effective.

## MATERIALS AND METHODS

### Cell lines and growth medium

BT-474 (Luminal B, ER^+^PR^+^HER2^-^), ZR751 (luminal A, ER^+^PR^-^HER2^-^), T47D, MCF7 (Luminal A ER^+^, PR^+^, HER2^-^), MDA-MB-231 and MDA-MB-468 (Basal, ER^-^, PR^-^, HER2^-^), MDA-MB-436 (Basal, ER^-^, PR^-^, HER2^-^, BRCA1-mutated 5396 +1 G>A in the splice donor site of exon 20, loss of nuclear BRCA1, does not express BRCA1 protein) [41] were from ATCC. SUM159PT (Basal, ER^-^, PR^-^, HER2^-^) was kindly provided by Dr. Ramesh Ganju, Ohio State University (OSU). BT-474 and ZR751 were grown in RPMI-1640, MDA-MB-231, MDA-MB-468, SUM159PT, and MDA-MB-436 were grown in DMEM. MCF7 was grown in EMEM+0.01 mg/ml bovine insulin. In all the mediums, 10% fetal bovine serum, and 100 IU penicillin/ml and 100 ng streptomycin/ml were added. Cells were cultured under a humidified atmosphere of 95% air/5% CO_2_ at 37°C. Cells were passaged weekly by treatment with 0.05% trypsin: 0.02% EDTA (w/v).

### Chemicals, antibodies, siRNAs, and primers

Reagents were obtained from MLN4924 (Adooq Biosciences), and cisplatin, Propidium iodide, RNase and BrdU (Sigma-Aldrich). Anti-y-H2AX (S139), pATR (S428), pATM (S1981), pChk1 (S345), pChk2 (T68) are from DNA damage antibody sampler kit from Cell Signaling Technology. Chk1, Chk2, Cdt1 (D10F11), p21, BRCA2 (D9S6V), and cleaved caspase 3 (D175) from Cell Signaling Technology. p27, Cdc25A (F-6), BRCA1, ATR (N-19), ATM, β-Actin, and GAPDH (G-9) are from Santa Cruz Biotechnology. APPBP1/NAE1, UBE1C/UBA3, Rad51, BrdU, and Anti-histone H3 phospho S10, are from Abcam. HRP conjugated Anti-mouse, Anti-rabbit, and Anti-goat secondary antibodies were obtained from GE Healthcare UK Limited. Fluorescent conjugated (Texas red and FITC) secondary antibodies were from Santa Cruz Biotechnology. BRCA1, BRCA2, and control siRNAs were from Dharmacon (ON-TARGET Plus siRNA, Dharmacon).

### Drug treatments

MLN4924 and cisplatin were dissolved in DMSO. For combination treatments MLN4924 was added before cisplatin treatment to activate the MLN4924-induced pathways. For 6 h treatment MLN4924 was added 2 h before cisplatin, and for 24 h treatment MLN4924 was added 6 h before cisplatin. For longer combination experiments using lower doses, MLN4924 was added 12–14 h before cisplatin.

### Cell viability assays, IC_50_, and combination index (CI) determination

Cell suspensions were seeded at 3,000–4,000 cells per well in 96-well plates in triplicates and incubated overnight at 37°C. Cells were treated with MLN4924, cisplatin, or DMSO at indicated doses for 86 h. For the combination experiment, MLN4924 was added 12–14 h before cisplatin. Cisplatin was added and cells were grown for an additional 72 h. Cell numbers were quantitated using the CellTitre-Glow Luminescent Cell Viability Assay Kit (Promega) based on ATP quantification. The combination index (CI) was calculated using the Chou Talalay method by using the Compusyn software [28]. The percent survival subtracted from 100% was indicative of the fraction affected (FA). Combination index values less than 1, equal to 1, and greater than 1, indicate synergism, additive, and antagonism, respectively.

### Colony formation assay

For the clonogenic assay, cells were seeded into 6-well plates (1000 cells/well). After 2 days, when the cells were 2–3 stage drugs were added, and after 12 days the colonies were fixed with 4% paraformaldehyde and stained with 0.05% crystal violet. The colonies with more than 50 cells were counted.

### SA-β-gal assay for senescence

The β-galactosidase staining assay was performed according to the manufacturer’s instruction (cell signaling senescence β-galactosidase kit), and blue color development was monitored under the microscope. Total and blue color cells were counted from at least 3–5 microscopic fields (~200 cells per field), and pictures were taken using the Nikon ELWD 0.3/OD75 microscope.

### Cell cycle arrest and Fluorescence-Activated Cell Sorting (FACS) analysis

Cells were treated with drugs, harvested, fixed in 70% ethanol at –20°C overnight, and stained with Propidium iodide (PI, 36 µg/ml) containing RNase (10 µg/ml) at 37°C for 15 min. Cells were then analyzed for cell cycle profile in a BD FACS Calibur flow cytometer (BD Biosciences). Data were analyzed using FlowJo software (FlowJo LLC).

### siRNA experiments

Cells were transfected 24 h with siRNA using lipofectamine TM2000 transfection reagent (Invitrogen) according to the manufacturer’s instruction. Drug treatments were conducted after 48 h of siRNA treatment. After drug treatment, cells were washed with PBS, followed by FACs analysis as described above.

### Immunoblotting

Immunoblotting was performed as described in [42, 43]. Total protein was extracted from the cells using sodium dodecyl sulfate (SDS) lysis buffer (50 mM Tris–HCl, pH 8.0, 10 mM EDTA, 1% SDS) with protease and phosphatase inhibitors followed by sonication. Protein concentrations were estimated using a Bio-Rad protein assay kit with BSA as standards. Western blot was performed following the Bio-Rad Western blot protocol using 4–15% Bio-Rad gradient gels. The membranes were cut based on the expected molecular weight of the protein and probed with specific antibodies. GAPDH or β-Actin were used as the loading control. WB using the LI-COR method was done following the protocol of LI-COR Biosciences. Briefly, after blocking with blocking buffer, the membrane was incubated with primary antibody overnight at 4°C. Following incubation with the appropriate secondary antibody (IRD-680 or IRD-800), the immunoreactive bands were visualized using LI-COR-Odyssey infrared scanner (LI-COR Biosciences).

### Immunofluorescence, BrdU labeling, and immunohistochemistry

Immunofluorescence was performed as described in [42, 43]. Briefly, cells were treated with drugs, fixed, and permeabilized with 2% paraformaldehyde in 0.5% Triton X-100 for 30 min. Coverslips were blocked with 20% serum in PBS for 1 h, stained with primary antibody overnight, followed by either Texas red (red), Alexa Fluor 594 (red), or FITC (green) conjugated secondary antibody. The coverslips were then mounted in Vectashield mounting medium with 0.25 μg/ml of 4′, 6-diamidino-2-phenylindole (DAPI) (Vector Laboratories). Fluorescence images were obtained with an Axioskop 40/Axioskop 40 FL fluorescence microscope equipped with N HBO103 and N HBO 75 microscope illuminating system and ZEN Software (Zeiss, Germany). Pictures were taken using AxioCam HRC (Zeiss) camera at 40× magnification. The digital images were processed with SPOT analysis software (Diagnostic Instruments). For BrdU labeling, cells were treated with 10 uM BrdU for 2 h at 37°C, washed with PBS (3 times 2 mins each). Cells were then fixed with 3.7% formaldehyde for 15 mins at room temperature, washed with PBS (3 times 2 mins each), and permeabilized with 0.1% Triton X-100 in PBS for 20 mins at room temperature. The DNA was denatured using 1N HCL for 10 mins on ice, and then with 2N HCL for 10 mins at room temperature. The cells were then washed with phosphate/citric acid buffer, pH 7.4 (182 ml of 0.2M Na_2_HPO4 and 18 ml 0.1M citric acid), incubated 10 min at room temperature, washed with 0.1% Triton X-100 in PBS (3 times 2 mins each), followed by detection with anti-BrdU primary antibody and secondary antibody. H&E staining and Ki-67 Immunohistochemistry was done at the OSU pathology core services. Imaging was done using the VECTRA 2.0.8 Imaging system using 40× magnification and percent of Ki67 positive cells were counted using inform 2.1.1 software.

### *In vivo* xenograft experiments

All animal care and experimental procedures were carried out according to the protocol by the OSU animal care facility. MDA-MB-231 (~5 × 10^6^ cells) in Matrigel (1:1) were injected subcutaneously into the dorsal flank of 8-week old NSG mice obtained from the Target Validation Shared Resources (TVSR) core facility, OSU. When the tumors reached approximately 150 mm^3^ in volume, the vehicle Hydroxypropyl beta-cyclodextrin (HPBCD) or drugs were injected. MLN4924 (30 mg/kg subcutaneously) was injected 6 h before cisplatin injection (both for MLN4924 alone and combination). Cisplatin was injected every alternate day (2 mg/kg for 3 days, followed by 1.5 mg/kg for 2 days, and 1.0 mg/kg for 2 days (intraperitoneally). Tumor size was measured on every fourth day and the tumor volume was calculated as follows: volume = longest tumor diameter × (shortest tumor diameter)^2^/2. The mice were sacrificed after 3 weeks, tumors were harvested, and the tumor tissues were collected and weighed. For histology, tissues were fixed in 4% paraformaldehyde and immune-histochemical analyses were done at the Pathology shared resources at the OSU.

### Statistical and bioinformatics analysis

Data are expressed as means ± SD of at least three independent experiments. Two groups were compared with a two-tailed Student’s *t*-test whereas multiple groups were analyzed with a two-way ANOVA to calculate the significance level and *p* < 0.05 was considered statistically significant. For comparison of gene expressions between TNBC and non-TNBC groups, the Curtis/METABRIC dataset was downloaded from Oncomine (https://www.oncomine.org).

## SUPPLEMENTARY MATERIALS


