# Supplementary material for: Both BRCA1-wild type and -mutant triple-negative breast cancers show sensitivity to the NAE inhibitor MLN4924 which is enhanced upon MLN4924 and cisplatin combination treatment
Source: Oncotarget. 2020 Feb 25;11(8):784–800. doi: 10.18632/oncotarget.27485 (PMC7055543; doi:10.18632/oncotarget.27485)
Supplement: Supplementary file 1 [file oncotarget-11-784-s001.pdf]

# Both BRCA1-wild type and -mutant triple-negative breast cancers show sensitivity to the NAE inhibitor MLN4924 which is enhanced upon MLN4924 and cisplatin combination treatment

SUPPLEMENTARY MATERIALS

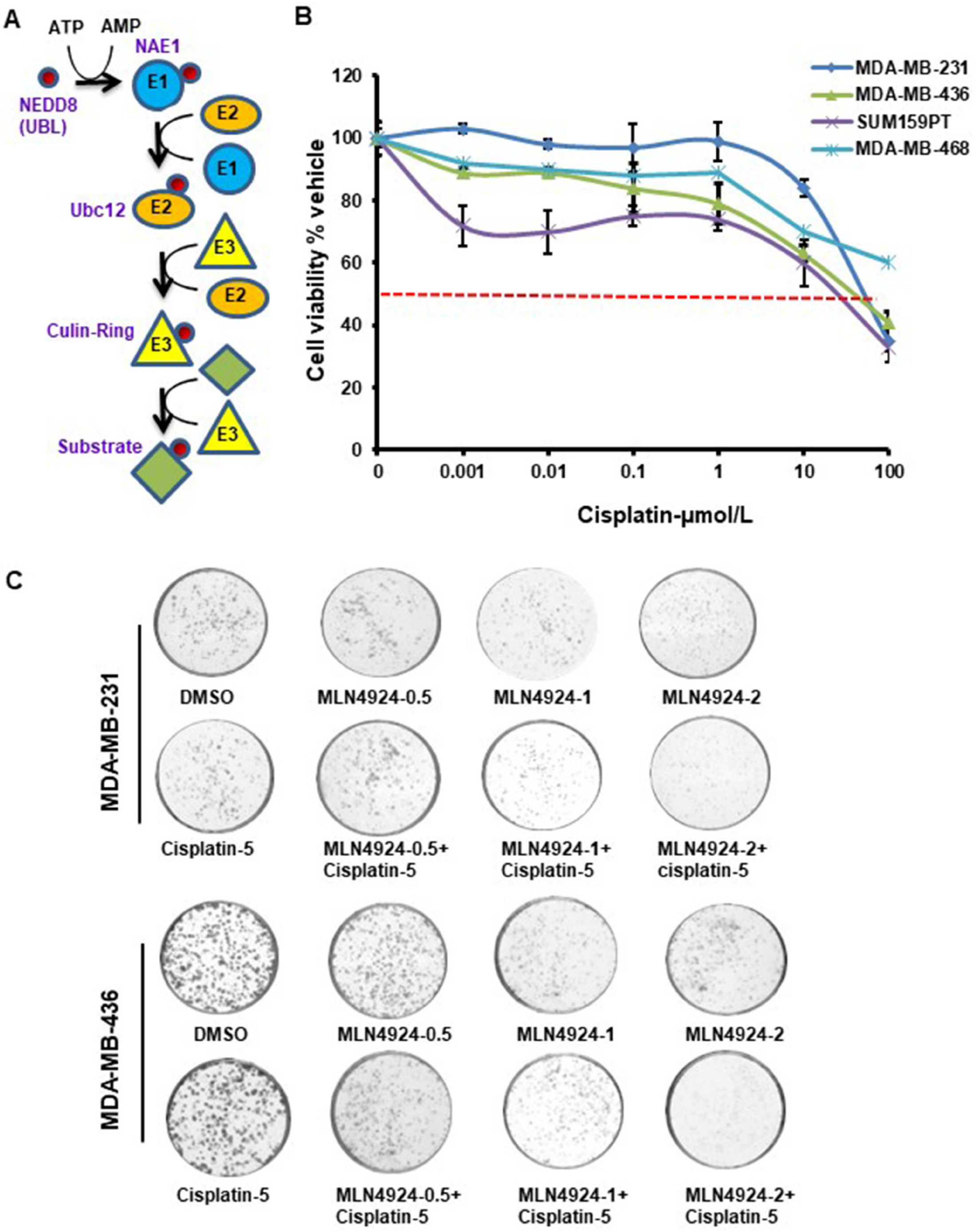

Supplementary Figure 1: (**A**) A schematic showing the neddylation pathway. (**B**) Cell survival upon cisplatin treatment. Cells were treated with cisplatin for 86 h. Data are expressed as mean ± SD of at least three independent experiments. (**C**) Colony formation upon using various doses of MLN4924, cisplatin, and MLN4924/cisplatin.

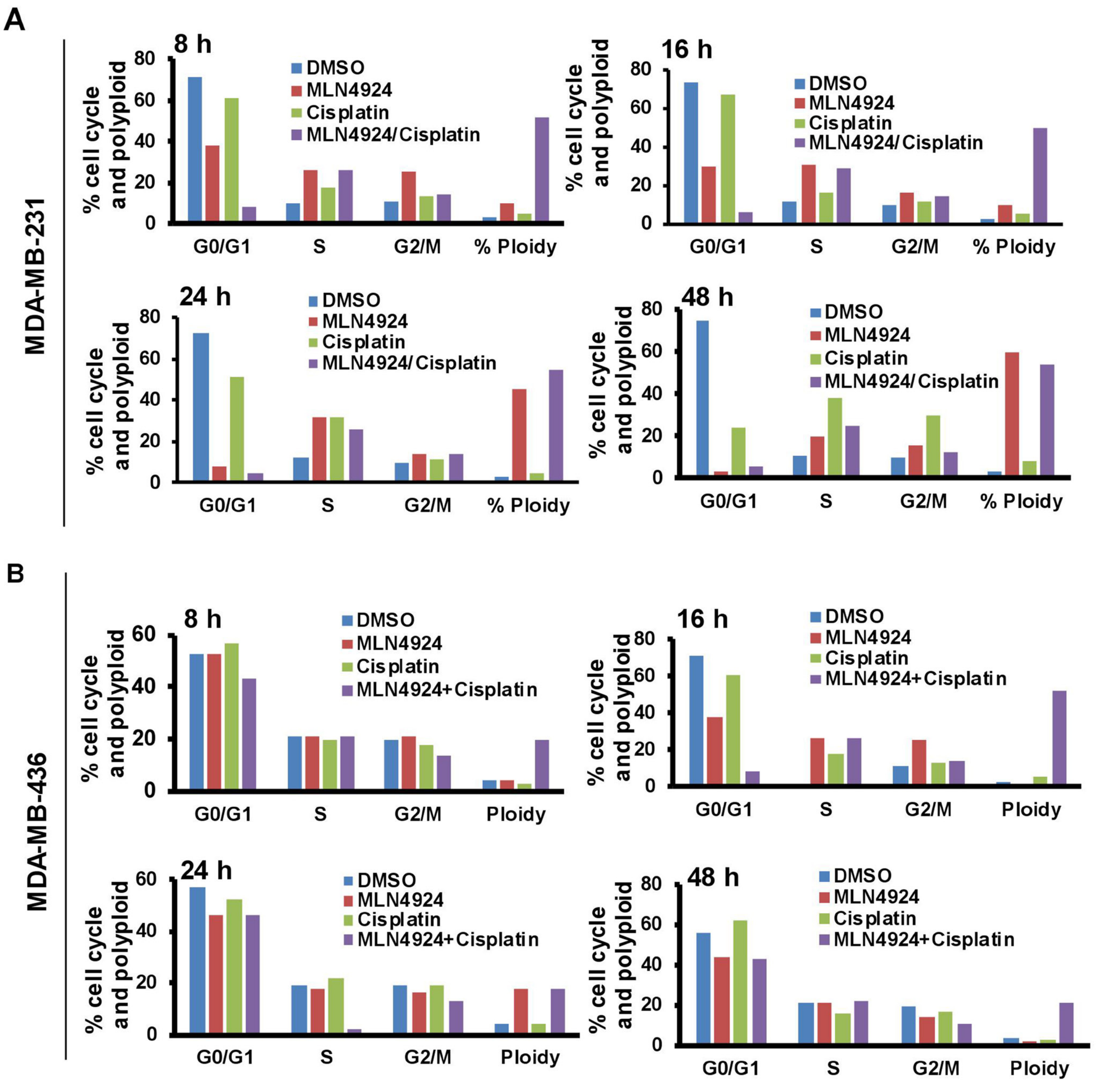

Supplementary Figure 2: Percent of cells in different cell cycle phases and percent of polyploid cells upon MLN4924, cisplatin, and MLN4924/cisplatin treatments. Data are from experiments described in Figure 5A. (**A**) MDA-MB-231. (**B**) MDA-MB-436.

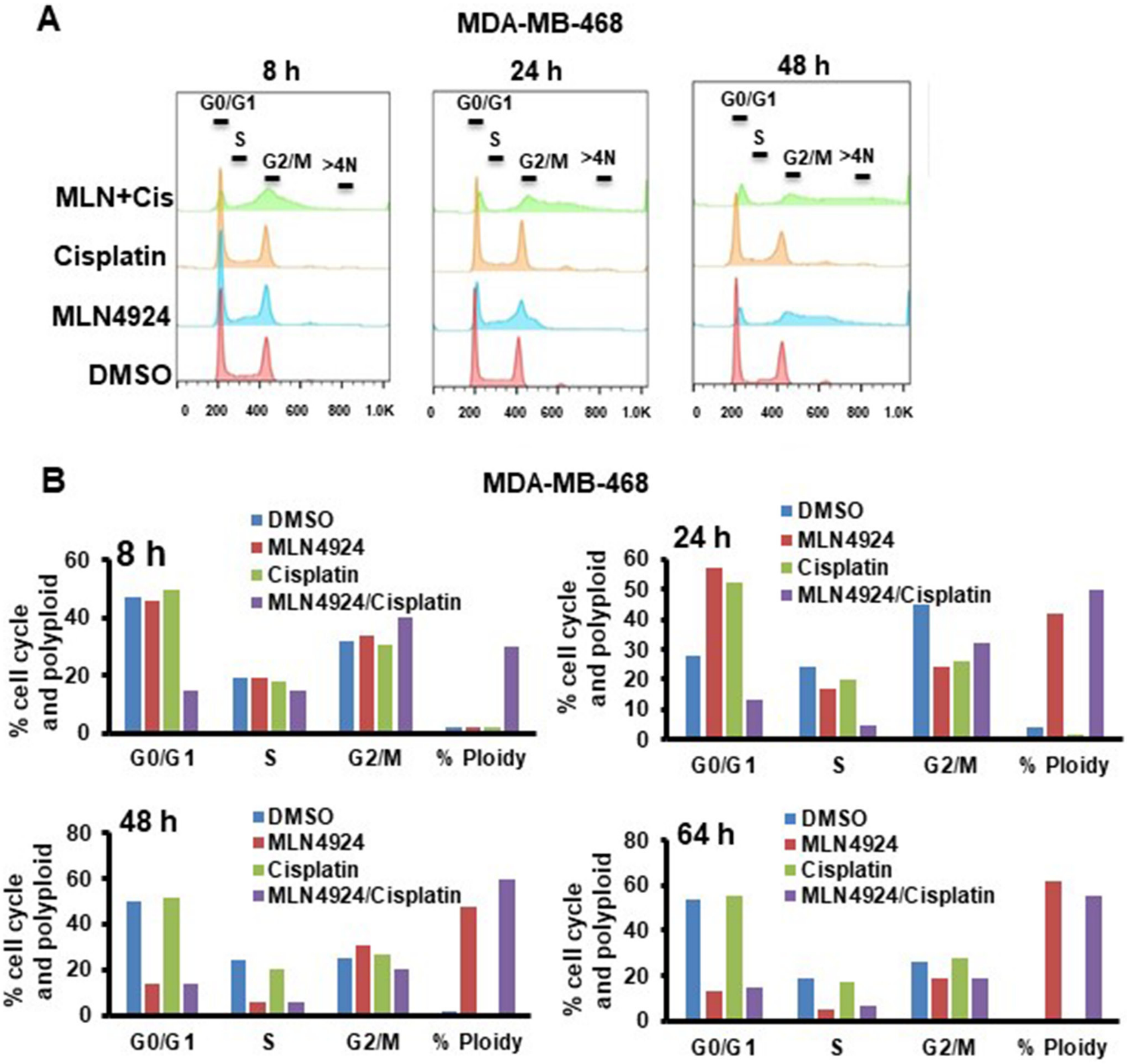

Supplementary Figure 3: (**A**) Cell cycle FACs analysis of MDA-MB-468 upon drug treatments showing the percent of cells in different cell cycle phases and percent of polyploid cells upon MLN4924 (2 uM), cisplatin (10 uM), and MLN4924/cisplatin treatments. Experiments were done as described in Figure 5A. (**B**) Data are from experiments described in A.

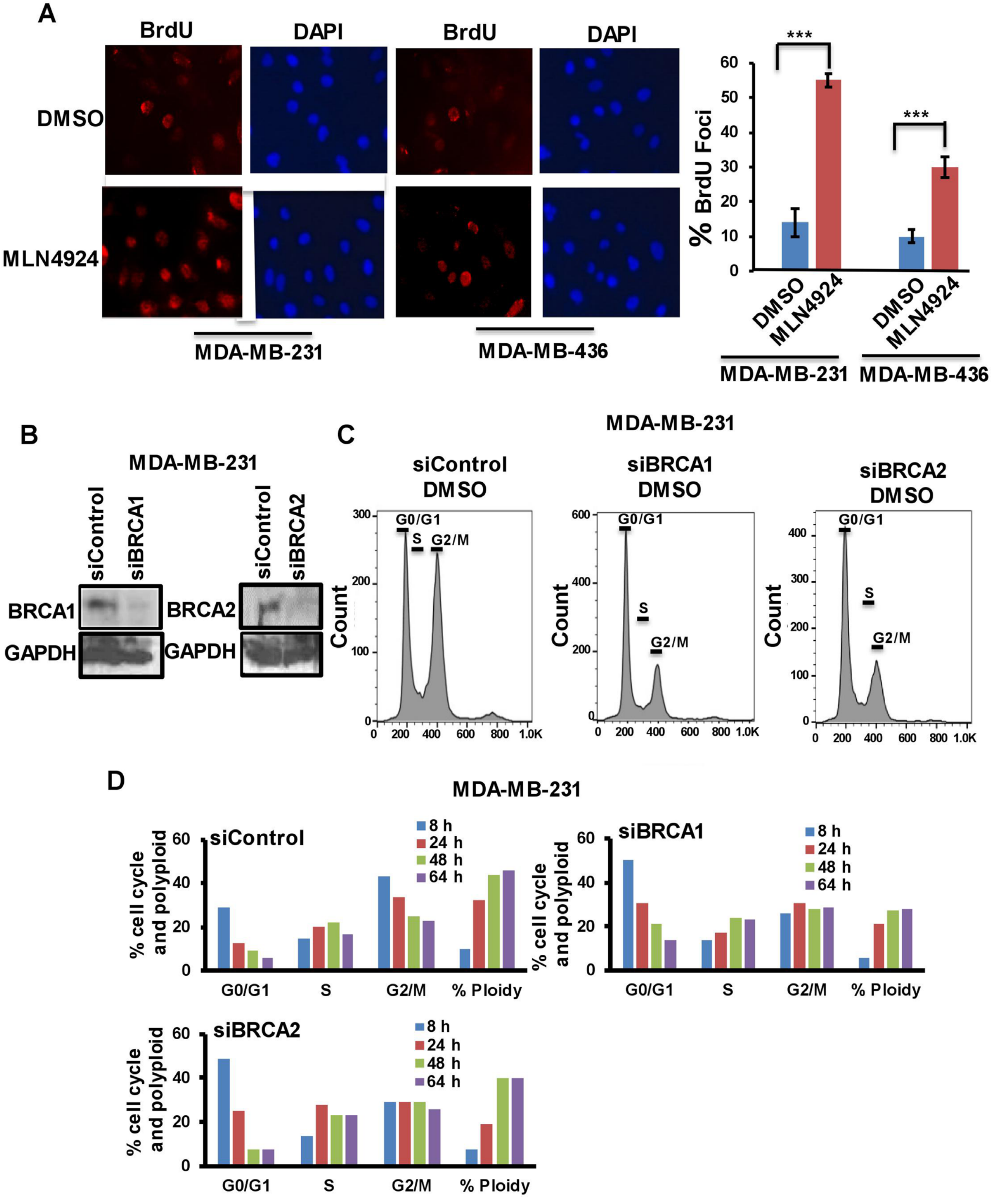

Supplementary Figure 4: (**A**) BrdU foci formation in MDA-MB-231 and MDA-MB-436 cells treated with vehicle and MLN4924 for 24 hours. Cells were treated with DMSO and MLN4924 (2 uM) for 48 h, BrdU labelling was done as described in methods, and IF was done with the BrdU antibody, and pictures were taken at 40X magnification. Several fields of cells (~50–100 cells/field) were counted to obtain the % BrdU foci number for each experiment. The results are from three independent experiments expressed as mean ± SD. $^{***}p < 0.0001$ indicates a significant difference. The left panel shows a representative field of BrdU foci containing cells and right panel shows the quantitation graph. (**B**) WB showing BRCA1 and BRCA2 knockdown. (**C**) FACs analysis of DMSO treated MDA-MB-231 cells as the control for Figure 5B. (**D**) Percent of cells in various cell cycle phases and showing polyploidy upon MLN4924, cisplatin, and MLN4924/cisplatin treatments from experiments described in Figure 5B.

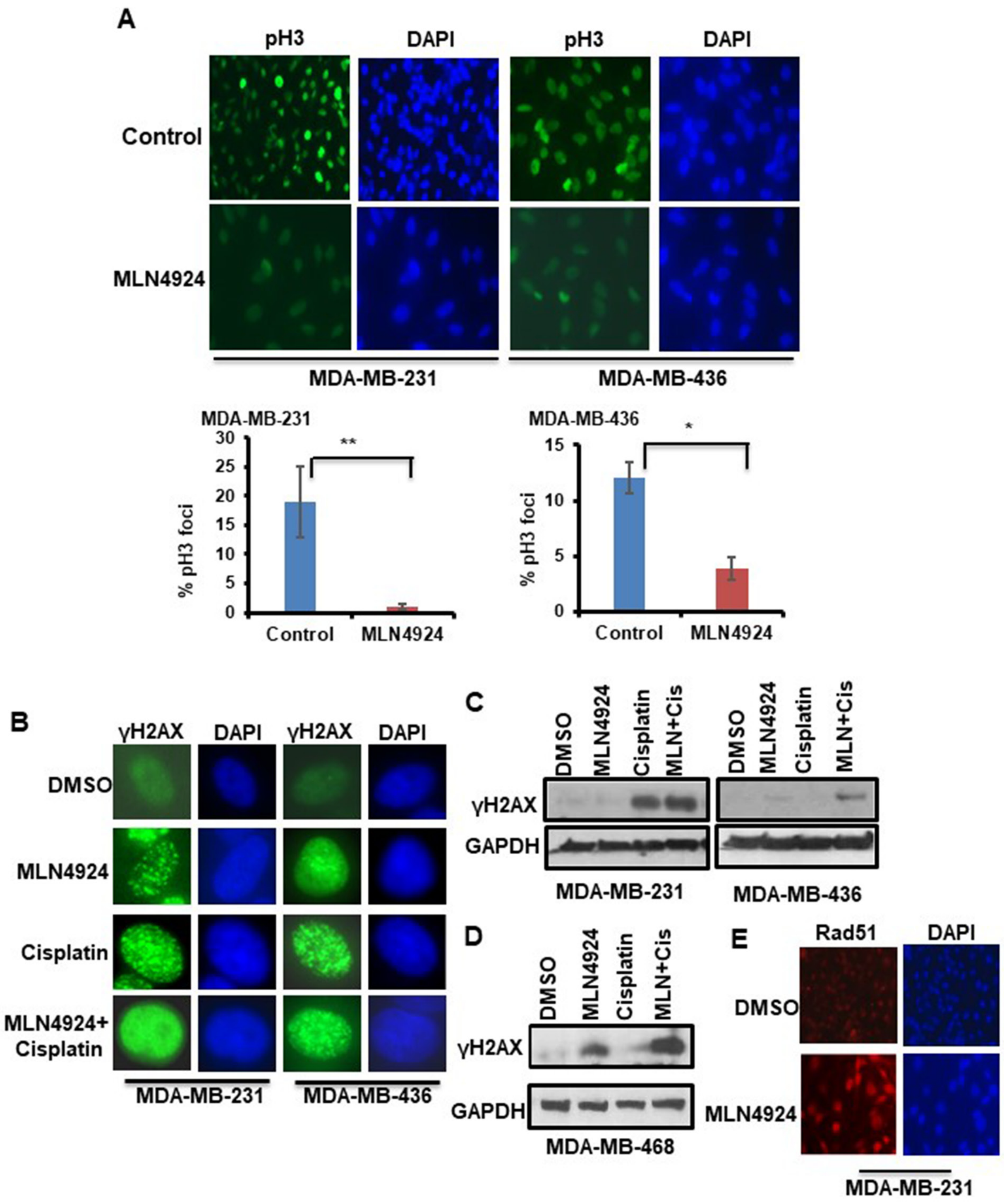

Supplementary Figure 5: (**A**) Phos-H3 foci formation is lower upon MLN4924 treatment compared to DMSO treatment indicating that cells do not progress to mitosis. Cells were treated with DMSO and MLN4924 (2 uM) for 48 h, IF was done with the phos-H3 antibody, and pictures were taken at 40X magnification. Several fields of cells (~50–200 cells/field) were counted to obtain the % phos-H3 foci number for each experiment. The results are from three independent experiments expressed as mean ± SD. $^{*}p < 0.01$ and $^{**}p < 0.001$ indicate a significant difference. The top panel shows a representative field of phos-H3 positive cells. (**B**) The yH2AX foci intensity was higher in MLN4924/cisplatin cotreated cells compared to the single treatments. A representative cell is shown from Figure 6B. (**C**) WB showing the yH2AX induction in MDA-MB-231 and MDA-MB-436 cells upon MLN4924 (2 uM), cisplatin (10 uM), and MLN4924/cisplatin treatments for 6 h. (**D**) WB showing the yH2AX induction in MDA-MB-468 cells upon MLN4924 (2 uM), cisplatin (10 uM), and MLN4924/cisplatin treatments for 24 h. (**E**) Rad51 foci formation in MDA-MB-231 cells upon MLN4924 (2 uM) treatment. Experiments were done as in Figure 6B.

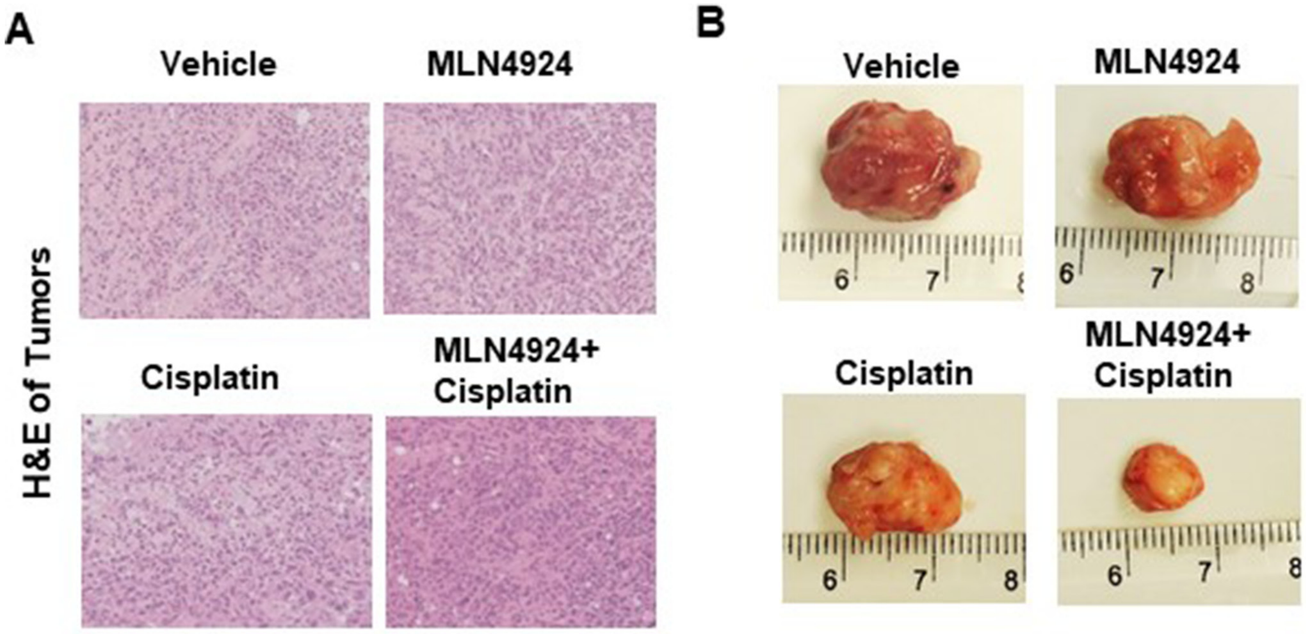

Supplementary Figure 6: (**A**) Representative images of tumor sections stained with Hematoxylin and Eosin (H&E). (**B**) Representative images of tumors from vehicle, MLN4924, cisplatin, and MLN4924/cisplatin groups.
